# RNA Profiling Analysis of the Serum Exosomes Derived from Patients with Chronic Hepatitis and Acute-on-chronic Liver Failure Caused By HBV

**DOI:** 10.1038/s41598-020-58233-x

**Published:** 2020-01-30

**Authors:** Jiajia Chen, Qingsheng Xu, Yan Zhang, Huafen Zhang

**Affiliations:** 1 State Key Laboratory for Diagnosis and Treatment of Infectious Diseases, National Clinical Research Center for Infectious Diseases, The First Affiliated Hospital, College of Medicine, Zhejiang University, Hangzhou, 310003 China; 2 Collaborative Innovation Center for Diagnosis and Treatment of Infectious Diseases, Hangzhou, 310003 China; 3 Department of Neurosurgery, The First Affiliated Hospital, College of Medicine, Zhejiang University, Hangzhou, 310003 China

**Keywords:** Hepatitis B, Hepatitis B, Diagnostic markers, Predictive markers

## Abstract

Hepatitis B virus (HBV) is the main causative viral agent for liver diseases in China. In liver injury, exosomes may impede the interaction with chromatin in the target cell and transmit inflammatory, apoptosis, or regeneration signals through RNAs. Therefore, we attempted to determine the potential functions of exosomal RNAs using bioinformatics technology. We performed RNA sequencing analysis in exosomes derived from clinical specimens of healthy control (HC) individuals and patients with chronic hepatitis B (CHB) and acute-on-chronic liver failure caused by HBV (HBV-ACLF). This analysis resulted in the identification of different types and proportions of RNAs in exosomes from the HC individuals and patients. Exosomes from the CHB and HBV-ACLF patients showed distinct upregulation and downregulation patterns of differentially expressed genes compared with those from the HC subjects. Gene Ontology and Kyoto Encyclopaedia of Genes and Genomes pathway analysis further confirmed different patterns of biological functions and signalling pathways in CHB and HBV-ACLF. Then we chose two upregulated RNAs both in CHB and HBV-ACLF for further qPCR validation. It confirmed the significantly different expression levels in CHB and HBV-ACLF compared with HC. Our findings indicate selective packaging of the RNA cargo into exosomes under different HBV attacks; these may represent potential targets for the diagnosis and treatment of HBV-caused liver injury.

## Introduction

Hepatitis B is a viral infection that attacks the liver and can cause both acute and chronic disease. The World Health Organisation estimates that, globally, 257 million people are living with hepatitis B virus (HBV) infection, which resulted in 887,000 deaths in 2015^1^. Chronic hepatitis B (CHB) includes three phases, i.e. immune-tolerant, immune-active, and inactive phases^2^. In the immune-active phase, various degrees of necroinflammation, with or without fibrosis, are observed in the liver^3^. Hepatitis flares can be controlled with antiviral therapy, but liver failure is a significant cause of overall morbidity, even with antiviral drugs^4^.

The median mortality rate associated with acute-on-chronic liver failure (ACLF) has been reported to range from 50% to 90%^5^, which is different from that with hepatitis flares. In China, the plasma exchange-centred artificial liver support system has improved the prognosis of HBV-caused ACLF (HBV-ACLF), but the 1-month mortality rate remains high at 38.4%^4^. A better understanding of the molecular mechanisms of the development and progression of immune-active CHB and HBV-ACLF may allow a more accurate prediction of their outcomes and the optimisation of therapeutic regimens. Therefore, reliable and effective diagnosis and treatment strategies are urgently needed.

Exosomes are spheroidal particles with a lipid bilayer membrane, containing various classes of nucleic acids, proteins, and lipids^6^. Exosomes have been recognised as universal intercellular communication vesicles released by cells and able to horizontally transfer biochemical signals from cell to cell, thereby affecting cell functions in both physiological and pathological conditions^7^. Exosomes contain different types of RNAs, both coding and non-coding. Coding RNAs include messenger RNAs (mRNAs), and non-coding RNAs (ncRNAs) include microRNAs (miRNAs), long noncoding RNAs (lncRNAs), and circular RNAs (circRNAs). Different cell types produce different quantities of RNAs under specific conditions, such as hypoxia, oxidative stress, and infections, which may modulate the host’s immune system^8,9^. In recent years, it has been revealed that exosomes and their contents participate in the human immune response caused by HBV. Hepatitis B viral components present in exosomes are transmitted into naïve human hepatocytes and NK cells, thus resulting in active infection and NK-cell dysfunction, with the latter partly attributed to the suppression of the nuclear factor-κB (NF-κB) and p38 mitogen-activated protein kinase (MAPK) signalling pathways^10^. Kouwaki *et al*.^11^ found increased levels of immunoregulatory miRNAs in exosomes from hepatocytes infected with HBV, which were then transferred to macrophages, thereby suppressing IL-12p35 mRNA expression in macrophages to counteract the host innate immune response.

LncRNAs found in circulating exosomes appear to be promising biomarkers, owing to their involvement in various biological processes, including critical steps in hepatic carcinoma development^12^. Susluer *et al*.^13^ found different lncRNA expression patterns in the plasma of patients (classified as chronic, inactive carriers, and resolved) with hepatitis B and predicted the roles of these RNAs. However, the expression patterns and roles of RNAs present in circulating exosomes in patients with HBV-caused liver injury are not well understood. Information on gene expression in serum exosomes of these patients could provide molecular clues about regulatory pathways and networks in different types of liver injury caused by HBV and help identify gene targets for diagnosis and therapy.

Thus, we investigated differentially expressed mRNAs, lncRNAs, and circRNAs in circulating exosomes from patients with CHB and ACLF using RNA sequencing to understand the molecular patterns of mRNAs and ncRNAs.

## Results

### RNA expression profiles in serum exosomes from healthy control (HC), CHB, and HBV-ACLF individuals

**Figure 1:**
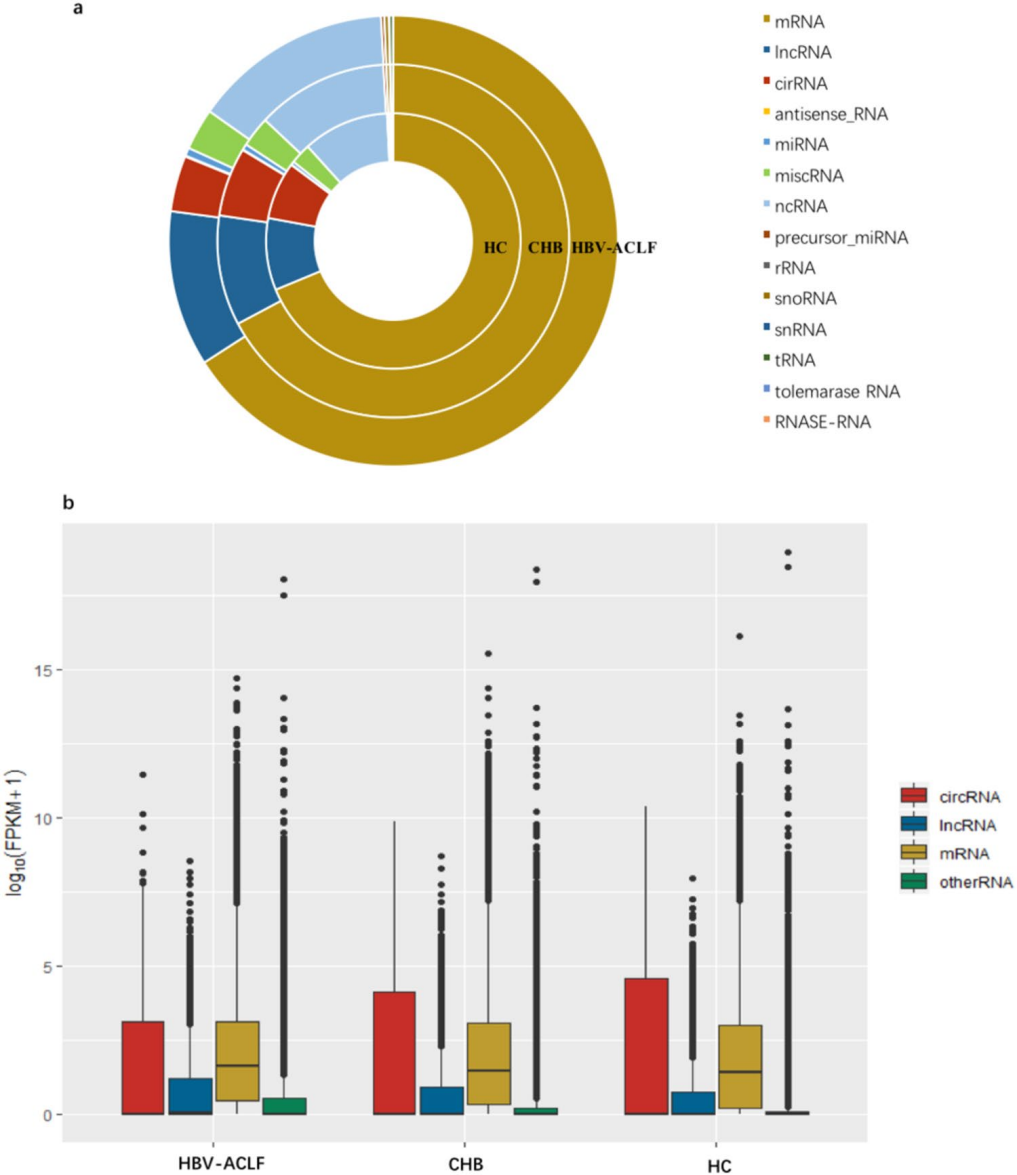
General gene expression profiles among CHB and HBV-ACLF patients and HC individuals. (**a**) Different types of RNAs were secreted into circulating exosomes (shown in different colours). The proportions of different types of genes were similar in each sample. (**b**) Log_10_(FPKM + 1) values of mRNAs, lncRNAs, circRNAs, and other RNAs are shown in the three groups.

By mapping RNA sequencing reads to the Genome Reference ConsortiumHuman genome build 38 (GRCh38_year_2014), we obtained the gene expression profiles of serum exosomes. A total of 21,022 (HC), 22,751 (CHB), and 24,436 (HBV-ACLF) expressed genes were identified, of which 15,593, 16,328, and 16,784 were coding genes in HC individuals and CHB and HBV-ACLF patients, respectively. Various types of RNAs, including mRNAs, lncRNAs, and circRNAs, were secreted into exosomes, and their proportions were similar among the three groups of samples (Fig. 1a). LncRNAs and mRNAs had higher expression levels in serum exosomes from the HBV-ACLF samples than in those from the HC and CHB samples. In contrast, circRNAs had lower expression levels in the HBV-ACLF patients than in the HC and CHB subjects (Fig. 1b).

**Figure 2:**
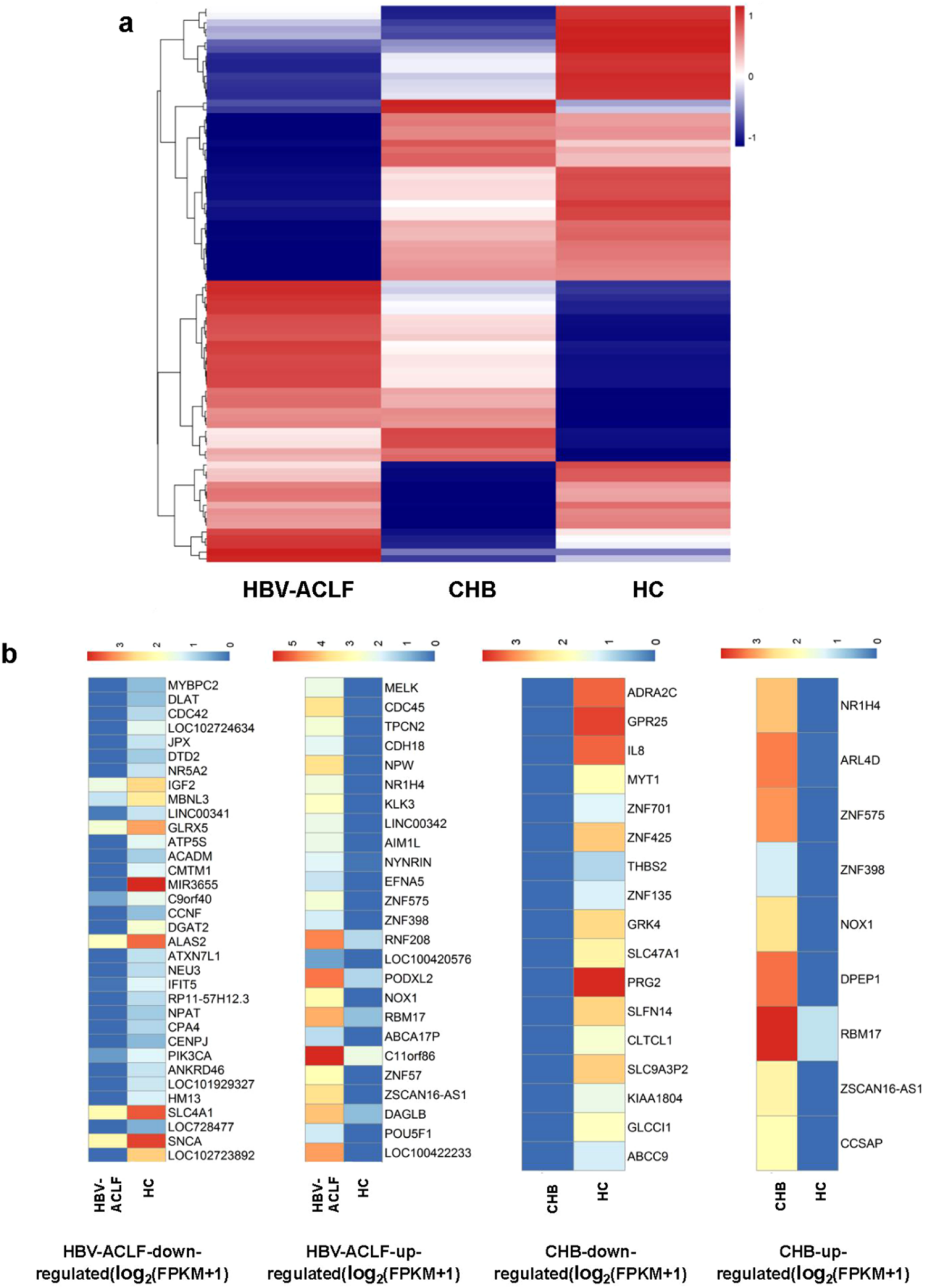
DEG profiles in circulating exosomes from the HBV-ACLF and CHB patients and HC individuals. (**a**) Heatmap of DEGs among the HBV-ACLF, CHB and HC samples. (**b**) Heatmaps of DEGs in the HBV-ACLF and CHB samples compared with those in the HC samples. Colours indicate log_2_(FPKM + 1) values, which ranged from low expression (blue) to high expression (red).

To obtain the differential expression profiles of serum exosomes from the HC, CHB, and HBV-ACLF individuals, we selected the differentially expressed genes (DEGs) by pairwise comparison (fold change, 2; p-value < 0.05) and plotted the data on heatmaps (Fig. 2a). As shown in Fig. 2b, compared with that in the HC samples, a distinct expression pattern of 9 upregulated genes and 17 downregulated genes was detected in the CHB patients (p < 0.05), whereas 25 upregulated and 34 downregulated genes were found in the HBV-ACLF patients (p < 0.05). Among these DEGs, 3 and 19 upregulated genes and 17 and 34 downregulated genes were only expressed in CHB and HBV-ACLF, respectively. Meanwhile, six upregulated genes and one downregulated gene were expressed in both CHB and HBV-ACLF. Compared with that in the CHB samples, a distinct expression pattern of 12 upregulated and 12 downregulated genes was found in the HBV-ACLF samples. All these DEGs may potentially provide a panel for the differentiation of HC, CHB, and HBV-ACLF samples.

### Gene Ontology (GO) and Kyoto Encyclopaedia of Genes and Genomes (KEGG) pathway analysis

**Figure 3:**
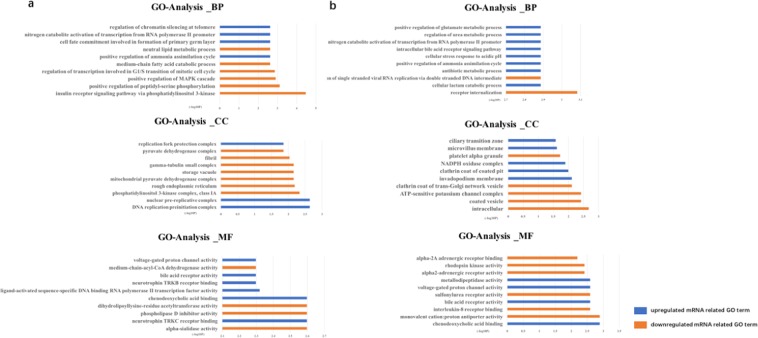
GO enrichment analysis of the differentially expressed exosomal mRNAs in the HBV-ACLF and CHB samples. (**a**) Top 10 significantly enriched GO terms, including biological process (BP), cellular component (CC), and molecular function (MF), in HBV-ACLF. (**b**) Top 10 significantly enriched GO terms, including BP, CC, and MF, in CHB.

GO functional analysis revealed differences in enriched GO categories among the three groups of samples. All filtered mRNAs were included in the GO analysis. Figure 3 shows the top 10 GO categories for the differentially expressed exosomal mRNAs (p < 0.05).

The downregulated exosomal mRNAs were related to 45 biological processes (BPs) in the CHB patients. The processes included receptor internalisation, the adenylate cyclase-inhibiting adrenergic receptor signalling pathway, negative regulation of uterine smooth muscle contraction, activation of MAPK activity via the adrenergic receptor signalling pathway, a defence response to nematodes, epidermal growth factor-activated receptor transactivation by a G protein-coupled receptor signalling pathway, desensitisation of the G protein-coupled receptor protein signalling pathway, signal transduction, regulation of the cytokine biosynthetic process, etc. Six downregulated genes (*GRK4*, *GPR25*, *ADRA2C*, *IL8*, *CLTCL1*, and *ABCC9*) were involved in signal transduction.

There were 53 BPs relevant to mRNAs that were upregulated in circulating exosomes of the CHB patients. The top four BPs included positive regulation of the ammonia assimilation cycle, the cellular stress response to acidic pH, the cellular lactam catabolic process, and the antibiotic metabolic process. CXCL8, NOX1, and NR1H4 were involved in 14, 16, and 19 BPs, respectively.

Four downregulated mRNAs (*GRK4*, *SLFN14*, *KIAA1804*, and *ABCC9*) were associated with ATP binding in molecular function (MF). Five upregulated genes (*DPEP1*, *NOX1*, *NR1H4*, *ZNF575*, and *ZNF398*) were associated with metal ion binding in MF. Five downregulated genes (*ZNF135*, *ZNF701*, *ZNF425*, *IL8*, and *KIAA1804*) were associated with ‘intracellular’ in cellular component (CC)^14^. Two upregulated genes (*DPEP1* and *NOX1*) were associated with ‘cell projection’ in CC, and six upregulated genes (*DPEP1*, *ZNF575*, *ARL4D*, *RBM17*, *NR1H4*, and *ZNF398*) were associated with ‘nucleus’ in CC.

In HBV-ACLF, 202 BPs were associated with the downregulated exosomal mRNAs. The top five BPs included the insulin receptor signalling pathway via phosphatidylinositol 3-kinase (PI3K), positive regulation of peptidyl-serine phosphorylation, the small-molecule metabolic process, glucose metabolic process, and positive regulation of protein serine/threonine kinase activity. There were 78 BPs relevant to the upregulated mRNAs. The top five enriched BPs included the pre-replicative complex assembly involved in nuclear cell cycle DNA replication, mitotic DNA replication preinitiation complex assembly, positive regulation of the ammonia assimilation cycle, cellular stress response to acidic pH, and cell fate commitment involved in the formation of the primary germ layer.

Three downregulated transcripts (*ALAS2*, *DLAT*, and *DGAT2*) were associated with ‘transferase activity, transferring acyl groups’ in MF, whereas *SNCA* and *NR5A2* were associated with phospholipid binding. Eight upregulated genes (*DAGLB*, *NOX1*, *NR1H4*, *ZNF57*, *RNF208*, *ZNF398*, *ZNF575*, and *CDH18*) were associated with metal ion binding in MF. *RBM17*, *ZNF57*, *ZNF398*, *ZNF575*, and *NYNRIN* were associated with nucleic acid binding in MF. Seven downregulated genes, (*SNCA*, *ALAS2*, *GLRX5*, *DLAT*, *DGAT2*, *ACADM*, and *ATP5S*) were related to ‘mitochondrion’ and ‘mitochondrial matrix’ in CC.

KEGG pathway enrichment analysis was performed for serum exosomal DEGs to understand the related pathways and molecular interactions in different states of necroinflammation. The data showed that the important pathways in CHB included the chemokine signalling pathway and endocytosis, which were associated with downregulated mRNAs, whereas asthma and the Notch signalling pathway were associated with upregulated transcripts. The 14 KEGG pathways associated with mRNAs differentially expressed in serum exosomes of the HBV-ACLF patients included leucocyte transendothelial migration, pancreatic cancer, renal cell carcinoma, bacterial invasion of epithelial cells, proteoglycans in cancer, prostate cancer, Fc gamma R-mediated phagocytosis, carbon metabolism, glycan degradation, and VEGF, Rap1, Ras, HIF-1, and T-cell receptor signalling pathways. PIK3CA and CDC42 were related to 12 and 10 of the above pathways, respectively.

Seven signalling pathways were greatly suppressed in the CHB samples (p < 0.05), whereas 20 pathways were suppressed in the HBV-ACLF samples; among these, only one was shared with the CHB samples, suggesting different inhibitory effects in CHB than in HBV-ACLF. And, the inhibitory effects on exosomal signalling pathways were more complex in HBV-ACLF than in CHB, suggesting that the inhibition of cell activities differs between different types of HBV attack.

### Quantitative real-time PCR for RNAs verification

**Figure 4:**
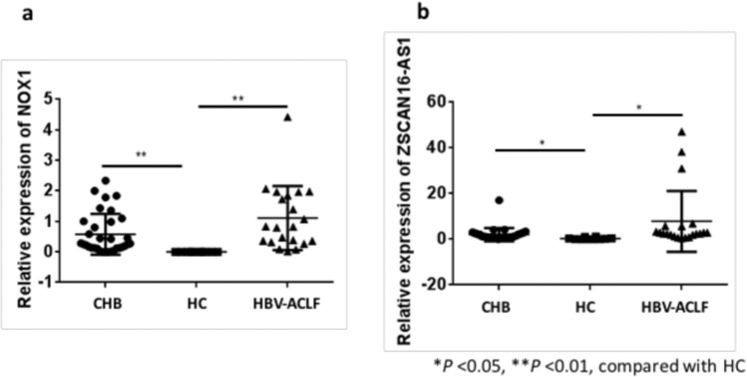
The fold changes of NOX1 mRNA (**a**) and lncRNA ZSCAN16-AS1 (**b**) from qRT-PCR results by using the 2^−ΔΔCT^ method.

Two upregulated RNAs (NOX1 mRNA and lncRNA ZSCAN16-AS1) both in CHB and HBV-ACLF were chosen to perform the qRT-PCR validation according to the sequencing results. When the HBV-ACLF and HBV patients compared to HC, the expression of NOX1 mRNA and ZSCAN16-AS1 both significantly increased (Fig. 4). The expression levels of NOX1 mRNA increased 5875-fold in patients with HBV-ACLF and 3057-fold in patients with CHB. But there was no significant difference between CHB and HBV-ACLF. The above results were in accordance with the initial screening study, and indicate these two RNAs in serum exosomes could potentially be used as a biomarker for HBV-ACLF or CHB.

### Correlation of RNAs with clinical parameters of CHB and HBV-ACLF patients

Although we did not find the significant difference of two RNAs between the CHB and HBV-ACLF patients, to investigate whether exosomal RNAs were related to the severity of HBV attack, we correlated these two RNAs’ expression levels with clinical indicators of HBV infection including serum HBV DNA levels, HBsAg levels, ALB levels, ALT levels, TBil levels, PT, PLT counts and MELD scores. The result showed that the levels of exosomal ZSCANA16-AS1 were negatively correlated with ALB (R = 0.279, *P* = 0.032) and positively with ALT (R = 0.313, *P* = 0.016). The levels of exosomal NOX1 mRNA were negatively correlated with ALB (R = 0.362, *P* = 0.004), PLT counts (R = 0.409, *P* = 0.001) and positively with ALT (R = 0.267, *P* = 0.036), TB (R = 0.356, *P* = 0.005) and MELD scores (R = 0.411, *P* = 0.001).

## Discussion

Exosomes can transfer various classes of RNAs, including mRNAs encoding specific proteins and ncRNAs of different sizes, such as lncRNAs, miRNAs, and circRNAs. Both coding RNAs and ncRNAs may act as protein cargos in exosomes, which transport proteins from one cell to another, impede the interaction with chromatin in the target cells, and modify gene expression. In this study, we revealed different expression profiles of RNAs in serum exosomes from healthy individuals and CHB and HBV-ACLF patients. Our data demonstrated the complexity of gene expression profiles in circulating exosomes and differences between CHB and HBV-ACLF. The descending order of the magnitude of circRNA expression was HC > CHB > HBV-ACLF. However, the expression levels of lncRNAs showed the opposite order. More DEGs were found in exosomes of the HBV-ACLF patients than in those of the CHB patients. Among DEGs we found the upregulated mRNA and lncRNA both differentially expressed in CHB and HBV-ACLF were NADPH oxidase 1 (NOX1) mRNA and ZSCAN16-AS1, which were confirmed by qPCR verification.

NOX1 are enzyme complexes with a membrane spanning catalytic NOX1 subunit, which transfer electrons from NADPH to molecular oxygen and generate superoxide to impact the cell fate^15^. NOXs are major sources of reactive oxygen species (ROS). As second messengers, NOX1-derived ROS plays an important role in gene expression, cell proliferation and migration in physiological conditions, and dysregulation of cell activation, cell death, mitogenic regulation and carcinogenesis in pathological conditions^14,16,17^. Recently more evidences demonstrated that NOX1 participates in the pathogenesis of cardiovascular diseases including atherosclerosis and hypertension, which are associated with the dysfunction of endothelia cells or vascular smooth muscle cells^18–20^. As a pro-inflammatory molecule, NOX1 may mediate oxidative stress by NF-κ B activation^21^. The NOX1 mRNA is constitutively expressed in the entire colon tissues and in the ileocecum at a lower level, whereas it is not detected in the esophagus and stomach^22,23^. But it was suggested that NOX1 may be one of the key molecules representing the initial trigger for host innate and immune response against *H. pylori* in stomach^24,25^. The main ROS-producing NOXs in the liver is NOX1, which is widely expressed by hepatocytes, hepatic stellate cell (HSC), and endothelial cells^26^. Oxidative stress is an important driving force in almost all chronic liver diseases. The NOX-derived ROS mediates hepatocyte apoptosis, HSC and endothelial cell activation in a spectrum of liver diseases. de Mochel *et al*.^27^ demonstrated that NOX1 was localized in the nuclei of hepatitis C virus-infected hepatocytes, which led to nuclear ROS production and hepatocyte injury. NOX1 expressed in HSC may improve liver fibrogenesis and proliferation. In fibrotic livers NOX1 was upregulated, and NOX1-knockout mice developed attenuated fibrosis after CCl4 injection^28^ or bile duct ligation^29^. In alcoholic liver diseases model, NOXs induced endothelin-1 in the liver sinusoidal endothelial cells, which can be attenuated by transfection of the p47phox siRNA^30^. NOX1 was detected in the plasma membrane, the endoplasmic reticulum, caveolae and endosomes of the cells^18,31,32^. It is not clear whether the effect of NOX1 function is determined by the levels of NOX1 and the location of ROS production.

Exosomal mRNA analysis showed that NOX1 mRNA was highly expressed in the serum exosomes in patients with CHB and HBV-ACLF, and the expression levels were positively correlated with inflammatory indexes of the progression of liver injury. It was demonstrated that NOX1 mRNA in exosomes may play a role in affecting the balance between ROS production and reduction. But it is not clear how and what exact role NOX1 mRNA in exosomes play in the development of the liver diseases caused by HBV. It may possibly induce hepatocyte apoptosis, activate Kupffer cells or initiate adaptive immune reaction because of exosomal transfer of ROS-related mRNA to the target cell. Or there may be extracellular way to generate ROS rather than non-phagocytic or phagocytic ways in cells. Further studies are need to confirm the relationship between exosomal NOX1 mRNAs and regulation of cell function.

LncRNAs have been widely researched in the development and progression of cancer. Recently, researchers have focused on the association between functional lncRNAs and viral hepatitis. LncRNAs induced by HCV infection may regulate the expression of coding genes required for the replication of the virus or regulation of genes involved in the cellular antiviral response^33^. The expression levels of the lncRNA maternally expressed gene 3 (MEG3)^34^ and the lincRNA p21^35^ have been found to be reduced during liver fibrosis and, thus, may potentially serve as biomarkers of fibrosis. The lncRNA growth arrest-specific transcript 5 (GAS5) inhibits HCV replication^36^ and participates in the activation and proliferation of HSCs^37^. In our study, a new exosomal lncRNA, ZSCAN16-AS1, increased significantly in serum exosomes of CHB and HBV-ACLF samples. They were also positively correlated with the severity of liver injury, including the indicators like ALT levels, TB levels and MELD scores. That may suggest the role of lncRNA in the mechanism of liver diseases caused by HBV. Then many new exosomal lncRNAs, other than ZSCAN16-AS1, were identified in CHB or HBV-ACLF samples. The lncRNA JPX has been found to activate the expression of the lncRNA X-inactive specific transcript (Xist), which affected cell proliferation and metastasis in HCC^38,39^. Linc00341 suppresses the VCAM1 expression and inhibits monocyte adhesion, which indicates that linc00341 has an anti-inflammatory function in endothelial cells^40^. However, most of these lncRNAs have not been previously reported or studied in liver injury^13^. Further research on these lncRNAs will provide clearer insights into the pathophysiological mechanism of information transmission for HBV-caused liver injury through intercellular exosomes.

Among DEGs we also found the upregulated NR1H4 mRNA. The NR1H4 is a member of the nuclear hormone receptor superfamily and is highly expressed in the human liver^41^. NR1H4 regulates target genes controlling cholesterol and glucose metabolism^42^ by functioning as a bile salt sensor^43^. Upon activation, NR1H4 suppresses cholesterol 7 alpha-hydroxylase (Cyp7a1), the rate-limiting enzyme in the classic or neutral bile acid synthesis pathway^44^. NR1H4 was found to be upregulated in exosomes of both CHB and HBV-ACLF patients. It revealed that, in exosomes, NR1H4 might participate in pathophysiological processes involved in liver damage caused by HBV. However we did not perform further validation.

Overall, analysis of circulating exosomal RNAs unveiled some potential biomarkers for the diagnosis of CHB and HBV-ACLF and provided clues about the possible mechanisms of different types of liver injury caused by HBV. Further investigations on the roles of exosomal RNAs in CHB and HBV-ACLF may potentially help in exploring the pathological mechanism for a more appropriate diagnostic and therapeutic methods for these patients.

## Methods

### Study participants Information

The standards used for immune-active CHB and HBV-ACLF were in accordance with the Update on Prevention, Diagnosis, and Treatment of Chronic Hepatitis B: AASLD 2018 Hepatitis B Guidance^3^ and the Guideline for Diagnosis and Treatment of Liver Failure^45^. The characteristics of the enrolled subjects for sequencing group and validation group are listed in Table 1.

**Table 1:** Characteristics of the enrolled patients and healthy control individuals (Mean ± SD).

Clinical parameter	Sequencing group			Validation group		
	immune-active chronic hepatitis B (n = 6)	HBV-caused acute-on-chronic liver failure (n = 6)	healthy control (n = 6)	immune-active chronic hepatitis B (n = 29)	HBV-caused acute-on-chronic liver failure (n = 21)	healthy control (n = 21)
Gender (Female/male)	2/4	0/6	2/4	8/21	1/20	11/10
Age (yr)	37.5 ± 13.6	41.8 ± 6.2	27.2 ± 5.2	35.1 ± 8.4	45.6 ± 12.7	28.3 ± 2.6
HBeAg (+/−)	5/1	3/3	—	21/8	9/12	—
HBV-DNA (IU/ml)	(2.13E + 07) ± (2.16E + 07)	(4.62E + 05) ± (4.89E + 05)	—	(1.32E + 08) ± (2.48E + 08)	(1.72E + 08) ± (6.06E + 08)	—
ALT (U/L)	469 ± 450	1381 ± 564	21 ± 16	494 ± 331	450 ± 372	24 ± 21
TB (*μ*mol/L)	35.0 ± 30.0	299.5 ± 94.0	9.3 ± 4.6	34.1 ± 26.6	334.5 ± 149.5	10.5 ± 4.13
sCr (*μ*mol/L)	70.2 ± 14.7	52.5 ± 5.7	67.5 ± 18.5	67.5 ± 12.2	80.8 ± 35.0	64.7 ± 14.3
PT (s)	13.3 ± 1.0	28.8 ± 8.4	10.9 ± 0.6	13.9 ± 1.8	26.0 ± 12.4	10.7 ± 0.5
WBC (×10^9^/L)	4.78 ± 1.67	6.67 ± 0.86	6.00 ± 0.98	4.90 ± 0.96	8.68 ± 3.60	6.37 ± 1.34
PLT (×10^9^/L)	166 ± 72	167 ± 77	266 ± 69	177 ± 61	133 ± 60	247 ± 64
HB (g/L)	140 ± 17	133 ± 17	140 ± 23	141 ± 17	137 ± 22	141 ± 21
MELD score	8 ± 3	22 ± 4	0 ± 3	8 ± 5	24 ± 6	0 ± 3

ALT: Aminotransferase; TB: Total Bilirubin; sCr: serum Creatinine; PT: Prothrombin Time; WBC: White Blood Cell Count; PLT: Platelet Count; HB: Hemoglobin; MELD score: Model for End-Stage Liver Disease score.

### Sample preparation and isolation and identification of exosomes

The serum samples were obtained when the patients admitted to the hospital, prior to further treatment. Serum samples were individually processed for the HC, CHB, and HBV-ACLF clinical cohorts. Each serum samples (4 mL) were obtained from each cohort. Exosomes were isolated from the serum samples using the ExoQuick exosome precipitation kit (System Biosciences, CA, USA), following the manufacturer’s instructions. Exosomes were solubilized in RIPA buffer (Thermo) and separated by polyacrylamide gel electrophoresis, transferred to a polyvinylidene fluoride membrane and probed with anti-CD63 antibody (EXOAB-63A-1; System Biosciences, CA, United States) according to the user manual. Exosomes were also analyzed under a transmission electron microscopy (JEM-1200EX; JEOL, Ltd., Japan).

### Total RNA extraction, RNA library construction, and sequencing

RNA was extracted from exosomes derived from human serum using TRIzol (Invitrogen, Carlsbad, CA, USA), according to the manufacturer’s instructions. The RNA concentration and purity were evaluated using a NanoDrop One spectrophotometer (NanoDrop Technologies, Inc., Wilmington, DE, USA). The extracted total RNA was stored at −80 °C until use. The sequencing group consisted of 6 CHB patients, 6 HBV-ACLF patients, and 6 HC, as detailed in Table 1. RNA libraries were constructed from 200 ng of total RNA using the total RNA-seq (HMR) library prep kit for Illumina (Vazyme Biotech Co., Ltd., Nanjing, China), following the manufacturer’s recommendations, including rRNA removal, ribosomal-depleted RNA fragmentation, reverse transcription to cDNA, terminal repair, adapter ligation, fragment sorting, uracil-DNA glycosylase treatment, and PCR amplification and purification steps. The library quality was assessed on the Agilent Bioanalyzer 2200 system (Agilent Technologies, Santa Clara, CA, USA). RNA sequencing of three groups six samples involved was performed on the Illumina HiSeq X Ten genome analyzer platform in the paired-end mode at Shanghai Yingbio Biotechnology Co., Ltd. (China). Raw sequencing data were evaluated using FAST-QC (http://www.bioinformatics.babraham.ac.uk/projects/fastqc/), including the quality scores across all bases, GC distribution over all sequence, etc. Accuracy of mapping was greater than 99.9% when quality score was greater than 30.

### Bioinformatics analysis

HISAT2^46^ was used to map clean sequencing reads to the human genome, which was downloaded from the UCSC (http://genome.ucsc.edu), Ensemble (http://asia.ensembl.org/index.html), and GenBank (http://www.ncbi.nlm.nih.gov/genbank/) genome databases. Then the reads distribution on gene structure and on chromosome were computed. Reads per kilobase per million mapped reads (RPKM) and fragments per kilobase of transcript per million mapped reads (FPKM) were adopted for standardisation of gene expression. DEGs were screened using the DESeq package (EMBL) between patients with HBV-ACLF or CHB and HCs. For the analysis, log_2_(fold change) >1 or ≤1, p value < 0.01 and a false discovery rate (FDR) <0.05 between two samples were used to identify DEGs and transcripts. Blast2G and TopGO were used to perform GO analysis. Pathway annotations of microarrayed genes were downloaded from KEGG (http://www.genome.jp/Kegg/). Pathway categories with FDR <0.05 were selected.

### Quantitative real-time PCR for differentially expressed RNA verification

The upregulated most differentially expressed mRNA and lncRNA both in CHB and HBV-ACLF were selected for verification. They were NOX1 mRNA and lncZSCAN16-AS1. The quantitative PCR validation group consisted of 29 CHB patients, 21 HBV-ACLF patients, and 21 HC, as detailed in Table 1. All the primers for quantitative PCR were synthesized by Sangon Biotech (Shanghai) Co., Ltd. The quantitative PCR reaction mixture contained 5 *μ*l of 2 × Master Mix (Roche), 0.3 *μ*l RNA-specific primer F (10 *μ*M), 0.3 *μ*l RNA-specific primer R (10 *μ*M), and double distilled nuclease-free water, which made a total volume of 9 *μ*l. Each 9 *μ*l mixture was added to a well in a 384-well PCR plate, and this was followed by addition of 1 *μ*l cDNA in each hole. The reaction conditions were as follows: 95 °C for 10 min followed by 45 cycles of 95 °C for 15 s and 60 °C for 60 s. The relative amount of RNA was normalized against an internal control, B2M, and the fold change in the amount of each RNA was calculated using the 2^−ΔΔCT^ method.

### Statistical analysis

The Statistical Package for Social Sciences for Mac (SPSS 25.0, Inc., Chicago, IL, USA) was used for the statistical analysis. Distributions were compared between two groups by the t-test for continuous variables, and the χ2 test for categorical variables. Distributions of the characteristics among three groups were compared by ANOVA. P < 0.05 was considered statistically significant.

### Ethical approval and informed consent

The research was approved by the Human Ethics Committee of the First Affiliated Hospital, School of Medicine, Zhejiang University. All methods were performed in accordance with the relevant guidelines and regulations. Informed consent was obtained from each patient included in the study.

## Data Availability

The datasets generated during and/or analysed during the current study are available from the corresponding author on reasonable request. Or Sequence ReadArchive accession is PRJNA562369.

## References

[1] World Health Organization. Hepatitis B. 2018. Available online at https://www.who.int/news-room/fact-sheets/detail/hepatitis-b (accessed on 18 July 2018).

[2] McMahon BJ (2009). The natural history of chronic hepatitis B virus infection. Hepatology..

[3] Terrault NA (2018). Update on prevention, diagnosis, and treatment of chronic hepatitis B: AASLD 2018 hepatitis B guidance. Hepatology..

[4] Chen JJ (2016). Plasma exchange-centered artificial liver support system in hepatitis B virus-related acute-on-chronic liver failure: a nationwide prospective multicenter study in China. Hepatobiliary Pancreat. Dis. Int..

[5] Jalan R, Williams R (2002). Acute-on-chronic liver failure: pathophysiological basis of therapeutic options. Blood Purif..

[6] Mateescu B (2017). Obstacles and opportunities in the functional analysis of extracellular vesicle RNA - an ISEV position paper. J. Extracell. Vesicles..

[7] Schiera G, Di Liegro CM, Di Liegro I (2015). Extracellular membrane vesicles as vehicles for brain cell-to-cell interactions in physiological as well as pathological conditions. Biomed. Res. Int..

[8] Eldh M (2010). Exosomes communicate protective messages during oxidative stress; possible role of exosomal shuttle RNA. PLOS ONE.

[9] Li L (2016). Exosomes derived from hypoxic oral squamous cell carcinoma cells deliver miR-21 to normoxic cells to elicit a prometastatic phenotype. Cancer Res..

[10] Yang Y (2017). Exosomes mediate hepatitis B virus (HBV) transmission and NK-cell dysfunction. Cell Mol. Immunol..

[11] Kouwaki, T., Okamoto, M., Tsukamoto, H., Fukushima, Y. & Oshiumi, H. Extracellular vesicles deliver host and virus RNA and regulate innate immune response. *Int J Mol Sci*. **18**(3) (2017). 10.3390/ijms18030666 PMC5372678 28335522

[12] Lee, Y. R. *et al*. Circulating exosomal non-coding RNAs as prognostic biomarkers in human hepatocellular carcinoma. *Int J Cancer*. (2018). 10.1002/ijc.31931 30338850

[13] Susluer SY (2018). Analysis of long non-coding RNA (lncRNA) expression in hepatitis B patients. Bosn. J. Basic. Med. Sci..

[14] Gimenez M, Schickling BM, Lopes LR, Miller FJ (2016). Nox1 in cardiovascular diseases: regulation and pathophysiology. Clin. Science..

[15] Lambeth JD (2004). NOX enzymes and the biology of reactive oxygen. Nat. Rev. Immunol..

[16] Szanto I (2005). Expression of NOX1, a superoxide-generating NADPH oxidase, in colon cancer and inflammatory bowel disease. J. Pathol..

[17] Tohru K (2009). Roles of Nox1 and other Nox isoforms in cancer development. Cancer Sci..

[18] Miller Francis J (2010). A differential role for endocytosis in receptor-mediated activation of Nox1. Antioxid. Redox Signal..

[19] Jagadeesha Dammanahalli K, Maysam T, Botond B, Bhalla Ramesh C, Miller Francis J (2012). Nox1 transactivation of epidermal growth factor receptor promotes N-cadherin shedding and smooth muscle cell migration. Cardiovasc. Res..

[20] Hilenski Lula L, Clempus Roza E, Quinn Mark T, Lambeth J (2004). David and Griendling Kathy K. Distinct subcellular localizations of Nox1 and Nox4 in vascular smooth muscle cells. Arterioscler. Thromb. Vasc. Biol..

[21] Marcela G, Brandon S (2016). M, Lopes Lucia R, Miller Francis J. Nox1 in cardiovascular diseases: regulation and pathophysiology. Clin. Science..

[22] Banfi B, Clark RA, Steger K, Krause KH (2003). Two novel proteins activate superoxide generation by the NADPH oxidase NOX1. J. Biol. Chem..

[23] Kazuhito R (2006). NADPH Oxidases in the Gastrointestinal Tract: A Potential Role of Nox1 in Innate Immune Response and Carcinogenesis. Antioxid. Redox Signal..

[24] Kawahara T (2005). Helicobacter pylori lipopolysaccharide activates Rac1 and transcription of NADPH oxidase Nox1 and its organizer NOXO1 in guinea pig gastric mucosal cells. Am. J. Physiol. Cell Physiol..

[25] Teshima S, Kutsumi H, Kawahara T, Kishi K, Rokutan K (2000). Regulation of growth and apoptosis of cultured guinea pig gastric mucosal cells by mitogenic oxidase 1. Am. J. Physiol. Gastrointest. Liver Physiol..

[26] Paik YH (2014). Role of NADPH oxidases in liver fibrosis. Antioxid. Redox Signal..

[27] de Mochel Nabora Soledad R (2010). Hepatocyte NAD(P)H oxidases as an endogenous source of reactive oxygen species during hepatitis C virus infection. Hepatology..

[28] Yong-Han P (2011). The nicotinamide adenine dinucleotide phosphate oxidase (NOX) homologues NOX1 and NOX2/gp91(phox) mediate hepatic fibrosis in mice. Hepatology..

[29] Wenhao C (2011). NOX1/nicotinamide adenine dinucleotide phosphate, reduced form (NADPH) oxidase promotes proliferation of stellate cells and aggravates liver brosis induced by bile duct ligation. Hepatology..

[30] Samantha Y, Hidekazu T, Kalra Vijay K (2009). Ethanol-induced expression of ET-1 and ET-BR in liver sinusoidal endothelial cells and human endothelial cells involves hypoxia-inducible factor-1 and microRNA-199. J. Immunol..

[31] Laurindo FR, Araujo TL, Abrahao TB (2014). Nox NADPH oxidases and the endoplasmic reticulum. Antioxid. Redox Signal..

[32] Manea A, Tanase Laurentia I (2010). Raicu Monica and Simionescu Maya.Transcriptional regulation of NADPH oxidase isoforms, Nox1 and Nox4, by nuclear factor-kappaB in human aortic smooth muscle cells. Biochem. Biophys. Res. Commun..

[33] Carnero E, Fortes P (2016). HCV infection, IFN response and the coding and non-coding host cell genome. Virus Res..

[34] Yu F, Geng W, Dong P, Huang Z, Zheng J (2018). LncRNA-MEG3 inhibits activation of hepatic stellate cells through SMO protein and miR-212. Cell Death Dis..

[35] Yu F (2017). Serum lincRNA-p21 as a potential biomarker of liver fibrosis in chronic hepatitis B patients. J. Viral Hepat..

[36] Qian X, Xu C, Zhao P, Qi Z (2016). Long non-coding RNA GAS5 inhibited hepatitis C virus replication by binding viral NS3 protein. Virology..

[37] Yu F (2015). Long non-coding RNA growth arrest-specific transcript 5 (GAS5) inhibits liver fibrogenesis through a mechanism of competing endogenous RNA. J. Biol. Chem..

[38] Sun S (2013). Jpx RNA activates Xist by evicting CTCF. Cell..

[39] Ma X (2017). X-inactive-specific transcript of peripheral blood cells is regulated by exosomal Jpx and acts as a biomarker for female patients with hepatocellular carcinoma. Ther. Adv. Med. Oncol..

[40] Huang TS (2017). LINC00341 exerts an anti-inflammatory effect on endothelial cells by repressing VCAM1. Physiol. Genomics..

[41] Zhang Y, Kast-Woelbern HR, Edwards PA (2003). Natural structural variants of the nuclear receptor farnesoid X receptor affect transcriptional activation. J. Biol. Chem..

[42] Trauner M, Claudel T, Fickert P, Moustafa T, Wagner M (2010). Bile acids as regulators of hepatic lipid and glucose metabolism. Dig. Dis..

[43] Cai SY, Xiong L, Wray CG, Ballatori N, Boyer JL (2007). The farnesoid X receptor FXRalpha/NR1H4 acquired ligand specificity for bile salts late in vertebrate evolution. Am. J. Physiol. Regul. Integr. Comp. Physiol..

[44] Bjursell M (2013). Ageing Fxr deficient mice develop increased energy expenditure, improved glucose control and liver damage resembling NASH. PLOS ONE..

[45] Liver Failure and Artificial Liver Group, C.S.o.I.D., CMA; Severe Liver Disease and Artificial Liver Group, Chinese Society of Hepatology, CMA. Guideline for diagnosis and treatment of liver failure. *Chin J. Clin Infect Dis*. **5**(6), 321–327 (2012).

[46] Kim D, Langmead B, Salzberg SL (2015). HISAT: a fast spliced aligner with low memory requirements. Nat. Methods..

